# The Bucharest international European Society for Organ Transplantation consensus on paediatric controlled donation after circulatory determination of death

**DOI:** 10.3389/ti.2026.16462

**Published:** 2026-06-30

**Authors:** Matthew J. Weiss, Marion J. Siebelink, Elena Cavazzoni, Maria A. Figini, Yasser Kazzaz, Silvio Nadalin, Thomas A. Nakagawa, Angie Scales, Beatriz Domínguez-Gil, Gabriel C. Oniscu, Umberto Cillo, Dominique E. Martin

**Affiliations:** 1 Transplant Québec, Montréal, QC, Canada; 2 Department of Pediatrics, Centre Mère-Enfant Soleil du CHU de Québec, Québec, QC, Canada; 3 University Medical Center Groningen Transplant Center, University Medical Center Groningen, Groningen, Netherlands; 4 Paediatrics and Child Health, University of Sydney, Sydney, NSW, Australia; 5 DonateLife, Sydney, NSW, Australia; 6 Pediatric Intensive Care Unit and Medical Coordination of Organ and Tissue Donation and Transplantation, Fondazione IRCCS Cà Granda, Ospedale Maggiore Policlinico of Milan, Milan, Italy; 7 Department of Pediatrics, Ministry of National Guards-Health Affairs, Riyadh, Saudi Arabia; 8 College of Medicine, King Saud Bin Abdulaziz University for Health Sciences, King Abdul Aziz Medical City, Riyadh, Saudi Arabia; 9 King Abdullah International Medical Research Centre, King Abdul Aziz Medical City, Riyadh, Saudi Arabia; 10 Department of General, Visceral and Transplant Surgery at University Hospital Tübingen, Tübingen, Germany; 11 Pediatric Critical Care Medicine University of Florida College of Medicine-Jacksonville, Jacksonville, FL, United States; 12 Honorbridge, Chapel Hill, NC, United States; 13 Paediatric Donation, NHS Blood and Transplant, Bristol, United Kingdom; 14 Organización Nacional de Trasplantes, Madrid, Spain; 15 Division of Transplantation Surgery, CLINTEC, Karolinska Institutet, Stockholm, Sweden; 16 Unità di Chirurgia Epatobiliare e Trapianto Epatico, Università degli Studi di Padova, Padova, Italy; 17 School of Medicine, Deakin University, Geelong, VIC, Australia

**Keywords:** donation after circulatory determination of death, donation system architecture, end-of-life care, ethics, paediatric

## Abstract

Paediatric controlled donation after circulatory determination of death (pcDCDD) is a well described pathway for deceased organ donation, but there has been wide variability in global uptake. There are substantial and differing barriers to pcDCDD across countries, including clinical, legal, and ethical issues. This process utilized a Delphi consensus methodology involving 30 international experts to develop recommendations to guide the development and operation of pcDCDD programs. Two survey rounds evaluated agreement on system requirements, donor identification, medical suitability, communication, end-of-life care, and ante-mortem interventions. Consensus recommendations emphasized the need for robust administrative and legal frameworks that explicitly support pcDCDD, multidisciplinary approaches for donor suitability assessment, and normalization of integrating donation into paediatric end-of-life care. The particularities of obtaining consent for both donation and antemortem interventions necessary for pcDCDD for patients that have never expressed a valid intent to donate were addressed. While our findings demonstrated international variability, strong consensus was obtained for multiple recommendations, suggesting the possibility of developing pcDCDD in varied international settings. The process also highlighted areas of knowledge gaps along the pcDCDD process that would benefit from sustained research.

## Introduction

**FIGURE 1:**
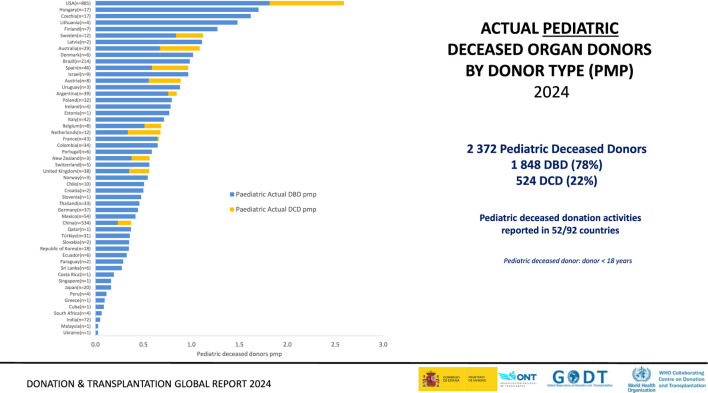
pcDCDD As a Portion of Total Peadiatric Deceased Donation.

**FIGURE 2:**
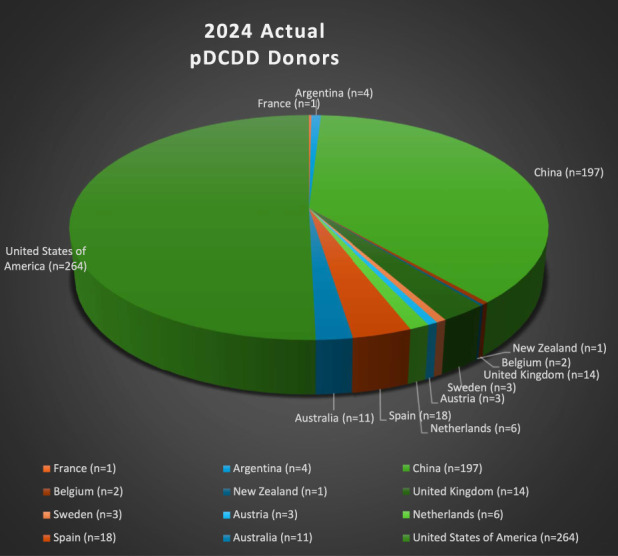
2024 Actual pDCDD Donors. Data provided by the GODT [1].

Controlled donation after circulatory determination of death (cDCDD) is the fastest growing form of deceased organ donation over the last 20 years internationally, but there has been substantial variation and acceptance of cDCDD between jurisdictions [1]. That variance is especially pronounced for potential paediatric donors. In the most recent report from the Global Observatory of Donation and Transplantation (GODT), only 12 countries reported at least one paediatric DCDD donor under 18 years of age, while 26 countries had at least one adult DCDD donor (Figure 1) [1]. The practice is also highly concentrated, with the United States and China accounting for 88% (461/524) of global pcDCDD activity reported to the GODT (Table 1). Outside of China and the US, some countries with lower donation volumes, such as the Netherlands, the majority of paediatric donors now come from the pcDCDD pathway. In most other countries, rates of pcDCDD lag behind their adult programs (Figure 2) [1, 2]. For example, in the UK from April 2024 to March 2025, 51% of adult donors were from the DCDD pathway, compared to 40% of paediatric donors, with conversion rates of potential adult DCDD donors double that of children (13% compared to 6.5%) [3, 4].

**TABLE 1:** Expert panel demographics.

​	Percent of participants
Participant demographics	Participants = 30
Age	
18–30 years	0%
31–40 years	16.7%
41–50 years	36.7%
51–60 years	36.7%
>60 years	10%
Gender	
Male	43.3%
Female	56.7%
Country of employment	
Australia	16.7%
Canada	3.3%
China	6.7%
Germany	3.3%
Italy	23.3%
Netherlands	6.7%
Saudi Arabia	3.3%
South Africa	3.3%
Spain	3.3%
United Kingdom	16.7%
United States	10%
United Arab Emirates	3.3%
Primary specialty	
Anaesthesiologist	2.9%
Ethicist	2.9%
Intensivist	40%
Nurse	14.3
Transplant surgeon	22.9%
Transplant or donation coordinator	14.3%
Others	2.9%
Primary patient population	
Paediatric medicine	63.3%
Both paediatric and adult medicine	36.7%
Not applicable to my role	0.00%
Adult medicine	0.00%
Primary sector of clinical practice for previous 12 months	
Public healthcare	93.3%
Private healthcare	3.3%
Both	3.3%
Not applicable	​
Type of hospital in which work	
Academic hospital	83.3%
Non-academic hospital	6.7%
Other academic centres and institutions	6.7%
Others	3.3%
Duration of experience with deceased donation or transplantation	
≥10 years	60%
5–9 years	30%
<5 years	10%
Author contributions to academic publications in deceased organ donation or transplantation	
Yes	66.7%
No	33.3%
Not applicable to my role	0%
Participation as a principal investigator in a clinical trial relating to deceased organ donation or transplantation	
No	66.7%
Yes	30%
Not applicable to my role	3.3%

The precise reasons for this variability have not been systematically investigated but likely involve a combination of factors such as paediatric mortality rates, professional attitudes about the integration of donation into end-of-life care, technical challenges relating to the recovery and implantation of small organs, consent/authorization rates, neuroprognostication, determination of death, and perceived ethical-legal concerns about pcDCDD [5–10]. Adult data suggests that compared to donation after neurologic determination of death (DNDD), the prolonged period between the decision to donate and withdrawal of life sustaining measures (WLSM) in the cDCDD pathway may contribute to lower consent rates [11]. Regardless of the particular contributing factors, the results are often similar: low referral and consent/authorization rates for potential pcDCDD donors, missed opportunities for families who may have wished to donate, and exacerbation of the organ shortage for recipients resulting in loss of life for transplant candidates [12–14].

There is limited evidence or procedural guidance available to conduct successful implementation of pcDCDD programs. National and international recommendations specific to pDCDD practice are rare and often adapted from adult recommendations [15–18], further impeding efforts to improve and expand pcDCDD activities. Consequently, the paediatric pathway for cDCDD was one of four topics explored as part of the European Society for Organ Transplantation (ESOT)’s international Consensus Project on cDCDD [19]. We report here on the results of the project that are specific to the paediatric population.

## Methods

The Delphi process applied in the overarching Consensus Project is outlined in detail in an accompanying manuscript [19]. A steering committee comprised of eight members with expertise in paediatric deceased donation developed a questionnaire addressing a range of topics identified as priorities following a literature review and group discussions. The committee identified expert panellists according to the criteria previously described, who were invited to participate in the Delphi process. The two coordinators were excluded from the expert panel.

Two survey waves were conducted using an online questionnaire administered by the independent company Adelphi Targis (Barcelona, Spain). All panellists who completed the first questionnaire were invited to complete the second wave survey. The questionnaire was refined for the second wave as previously described [19]. In responding to questions about pcDCDD, panellists were advised that paediatric donation encompassed donors aged between 37 weeks corrected gestational age (CGA) and 18 years.

In each survey round panellists indicated their level of agreement with a series of statements using a Likert scale (1-9, strongly disagree–strongly agree); responses were analysed using descriptive statistics with “disagreement” assigned to ratings 1–3, “neither agree nor disagree” 4-6, and “agreement” to ratings 7–9. Participants could alternatively indicate if a question was not relevant to their expertise. Responses from those who indicated they lacked relevant expertise were removed from the denominator when evaluating consensus on specific questions. Statements that reached 75% agreement were deemed to achieve consensus. The results from the second round relating to definition of terms are reported elsewhere [19].

## Results

Thirty experts from 12 countries completed the first round of the Delphi process, all of whom completed the second round. Panel demographics are shown in Table 1.

Consensus was achieved on 121 statements after the first round, and on 14 further statements after the second round. The 47 recommendations summarised in Tables 2–8 reflect the statements for which consensus was achieved, noting some were combined for efficiency.

**TABLE 2:** Consensus recommendations regarding baseline requirements for pcDCDD.

The following are required for the successful implementation of pcDCDD programs
1. Governments and health systems must establish a legal and ethical framework allowing for WLSM as part of routine paediatric end-of-life care, independently and prior to efforts to develop pcDCDD programs
2. Once WLSM legal and ethical frameworks are established, authorities must also establish a DCDD legal and ethical framework applicable to pediatric patients that includes: a. Determination of death by circulatory criteria; b. Integration of the offer of pcDCDD as part of routine end-of-life care when WLSM is a possibility; c. Establishing mechanism(s) for consent or authorization for pcDCDD; d. Establishing mechanism(s) for consent or authorization of *ante mortem* interventions; e. Criteria for consent for research relating to pcDCDD.
3. Ethical guidance to support healthcare professionals involved in decision-making about end-of-life care in the context of pcDCDD.
4. Inclusion of pcDCDD within the portfolio of governmental health authorities
5. Education for clinicians regarding clinical, ethical, and legal aspects of pcDCDD. In countries with pcDCDD programs: a. NICU and PICU staff should receive training specific to cDCDD, including training relating to donation discussions with families and donor suitability b. Organ donation staff should receive specific training on all elements of paediatric donation
6. Public education about deceased donation should include specific information pertaining to the pcDCDD pathway, to promote public understanding and awareness of how this may differ from other donation pathways
7. When evaluating the potential costs and benefits of introducing or expanding pcDCDD programs, careful attention should be given to the local context and the potential impact of legislation, available technologies, and cultural norms on opportunities for pcDCDD and utilisation of organs

pcDCDD Paediatric controlled donation after circulatory determination of death; WLSM, Withdrawal of life sustaining medical treatment; NICU, Neonatal Intensive Care Unit; PICU, Paediatric Intensive Care Unit.

**TABLE 3:** Consensus recommendations for the design of clinical protocols for pcDCDD.

1. Clinical triggers (tailored to local practices) should be developed to ensure timely a. Identification of all potential pcDCDD donors and routine communication of these cases to the relevant donation personnel, to assess their suitability for pcDCDD b. Consideration of organ donation options as part of decision-making about end-of-life care
2. Clinical triggers should include patients in whom death is considered inevitable following planned WLSM.
3. Identification of potential pcDCDD donors should be a recognized responsibility of a. Any healthcare professional caring for patients in a critical condition who are receiving life sustaining measures, and b. Intensive care and emergency medicine teams
4. Paediatric cDCDD protocols require guidance that addresses a. Criteria identifying possible donors whose medical suitability to donate should be evaluated b. When and to whom information about possible donors should be communicated for assessment of medical suitability for donation c. When and by whom patients and/or their substitute decision maker (SDM) should be contacted to discuss the possibility of pcDCDD. d. Care of the patient while initial assessments of suitability for donation and/or donation decision-making are made
5. Prior to WLSM in the context of pcDCDD, organ donation organization staff should meet to confirm or reconfirm: a. Introductions are made for all personnel involved in the donation process b. Roles and responsibilities of each staff member c. Donor paperwork and legal authorisations have been reviewed d. Planned organ retrieval and surgical steps including subsequent tissue retrieval e. Agreement on procedures if death does not occur in the timeframe f. Timing of WLSM. g. Finalisation of operating theatre requirements

pcDCDD Paediatric controlled donation after circulatory determination of death; WLSM, Withdrawal of life sustaining medical treatment.

**TABLE 4:** Consensus recommendations for the assessment of medical suitability for pcDCDD.

1. Guidance for the assessment of medical suitability for pcDCDD in individual cases should be established at the national level and should be informed by understanding of relevant factors that may vary between healthcare institutions (see Box 1)
2. When evaluating medical suitability for pcDCDD: a. Assessment of suitability to donate specific organs is recommended b. Individual characteristics of the potential donor as well as local healthcare system factors that may influence the probability of successfully recovering and utilising organ(s) in transplantation should be assessed
3. Criteria used in the assessment of individual suitability for pcDCDD should distinguish between potentially modifiable healthcare system factors that may influence the feasibility of pcDCDD and the clinical characteristics of individual patients that may influence the probability of death occurring within a specified time following WSLM (see Box 1)
4. Decisions about donor suitability in pcDCDD should not rely on a single method of prediction of time to death after WLSM because prediction by the clinical assessment of an attending intensivist or scoring systems (e.g., Dallas, CHLA) is imprecise and often unreliable
5. Individual evaluation of suitability for pcDCDD should be multi-disciplinary and involve the treating healthcare professionals, donation personnel and, if required, consultation with transplant teams
6. Where feasible, any child aged 37 weeks CGA or older should be considered a possible pcDCDD donor regardless of weight and relevant donation professionals should be consulted
7. Where there are logistic or other system constraints that mean it is not feasible to attempt pcDCDD, data should be collected and used to inform efforts to overcome barriers to pcDCDD in future
8. Where prioritisation in donation is necessary and feasible, donation of specific organs following pcDCDD should be prioritised in a manner that maximises the number of organs utilised and considers the needs of current candidates for transplantation. a. Where feasible, donation of paediatric hearts should be prioritised following pcDCDD, due to the severity of unmet needs for heart transplantation in the paediatric population and the difficulties of size matching

pcDCDD, Paediatric controlled donation after circulatory determination of death; WLSM, Withdrawal of life sustaining medical treatment.

**TABLE 5:** Consensus recommendations on communication about donation opportunities for pcDCDD.

1. It is the responsibility of the treating clinician with involvement of the healthcare team caring for a critically ill patient to communicate with the patient’s family about decisions regarding WLSM at the end of life
2. Where clinically relevant, information about potential opportunities for pcDCDD should routinely be provided to parents or other SDMs as part of end-of-life decision-making
3. In the context of pcDCDD, unless discussion of donation is raised independently by the child’s family, discussions should be **initiated** and led where possible by a trained paediatric donation professional who is not involved in the clinical care of the potential donor
4. Where possible, donation discussions should be led by a trained paediatric donation professional who is not involved in the clinical care of the potential donor
5. Communication with patients (where possible and appropriate), their parents and/or other SDMs should ensure that decisions about WLSM are not influenced by the possibility of organ donation a. Information about pcDCDD should only be proactively provided to patients (where possible and appropriate), their parents and/or other SDMs after a decision has been made about WLSM b. If the patient or their SDM request information about donation prior to finalization of WLSM decisions, they should be informed that donation may become a possibility that will be explored fully, but that the current care plan remains focused on active treatment and comfort for the child c. Formal decisions about pcDCDD resulting in lawful consent/authorization for donation should only be made after a decision has been made regarding WLSM.
6. The disclosure of information about pcDCDD protocols to a child’s parents or other SDM during decision-making about donation should satisfy the requirements for lawful consent/authorization in the relevant jurisdiction. a. The scope, depth, and detail of information about pcDCDD protocols disclosed during decision-making about donation should be tailored to the preferences of the patient, their parents and/or other SDM b. Detailed information about the process of organ removal or invasive *postmortem* interventions such as NRP should only be provided to patients, their parents or other SDM, by a trained donation specialist
7. When a child’s parents or other SDM is making a decision about pcDCDD, they should routinely be provided with some information about: a. Options for donated organs or tissues to be used in research if not utilised in transplantation b. The possibility of “stand down” (authorization without organ recovery), when an attempt at pcDCDD is aborted because death has not occurred within a timeframe that enables organ eligibility for transplantation, as defined by local protocols c. Potential *ante* mortem and *postmortem* interventions that may be used to increase the probability of successful donation for transplantation d. The potential implications of end-of-life care choices for pcDCDD, i.e., how some choices may influence the probability of successful recovery and utilisation of organs for transplantation
8. Organ donation organization staff overseeing or involved in pcDCDD for an individual may provide information or advice to the patient’s treating team regarding how end-of-life care choices (e.g., comfort care provision, mode of WSLM) may impact the likelihood of successful donation of organs for transplantation. a. If the child’s, parents or SDM’s preferences regarding end-of-life care limit the potential to recover organs via pcDCDD, they should be informed, if relevant, that it may not be feasible to support donation b. If the child’s, parents’ or SDM’s preferences regarding end-of-life care (e.g., timing or location of WLSM) may negatively impact opportunities for donation or utilisation of organs, it is important they understand that fewer organs may be able to be donated and successfully transplanted c. When the availability of surgical teams influences the optimal time for WLSM, the options and likely impact on donation and transplantation outcomes should be explained to the child’s parents or SDM who will make an informed choice regarding the timing of WLSM. d. When options are available for the location of WLSM that influence the likely success of donation of organs for transplantation, the child’s parents or SDM should make an informed choice regarding the location
9. In paediatric patients with devastating brain injury who do not meet criteria for neurological determination of death (NDD) at the time of donor referral, discussions about pcDCDD should include information about the possibility, where relevant, of donating via the NDD pathway

pcDCDD, Paediatric controlled donation after circulatory determination of death; WLSM, Withdrawal of life sustaining medical treatment

**TABLE 6:** Consensus recommendations for decision-making about donation and end-of-life care.

1. Guidance should be developed that establishes how decisions should be made about donation and end-of-life care by SDMs in accordance with relevant laws, e.g., making clear when the person’s known or estimated preferences should take priority in decision-making
2. When it is lawful to do so, and a child is (or was previously) competent to have made decisions or expressed values and preferences about donation or end-of-life care, their wishes should be respected where possible in decision-making
3. When it is lawful to do so, a child’s parents or other SDM should make end-of-life care decisions based on what they believe would be best for the child, all things considered
4. When making decisions about potential aspects of end-of-life care that may influence the likelihood of successful donation of organs following pcDCDD, the known or estimated preferences of the child should determine choices where possible
5. If a child’s parents or other SDM believes that what is best for the child conflicts with what the child would decide if they were able to make their own decision about pcDCDD, the parents or other SDM should make the decision they believe the child would make, provided this approach is lawful and the child is of sufficient maturity

pcDCDD, paediatric controlled donation after circulatory determination of death; WLSM, withdrawal of life sustaining medical treatment; SDM, substitute decision maker.

**TABLE 7:** Consensus recommendations on the use of ante mortem interventions for pcDCDD.

1. The potential benefits of using *ante mortem* interventions should be considered inclusive of their potential impact on transplant recipients, on fulfilment of a child’s known or estimated goal to donate organs for transplantation, and on the child’s family a. When evaluating the potential benefits of using an *ante mortem* intervention, the potential benefits of not using the intervention, such as achievement of a patient’s wish to avoid clinical procedures at the end of life should also be considered
2. Where available, lawful, and clinically relevant, use of *ante mortem* interventions should be considered if their potential benefits outweigh their potential burdens or risks in a child for whom donation is known or estimated to be a goal
3. *Ante mortem* interventions should only be considered where there is a clear clinical justification for their potential to optimise donation and transplant
4. Where required, analgesia and/or sedation should be provided for *ante mortem* interventions for donation as they would be for the same interventions if performed for therapeutic purposes
5. Research should be undertaken to provide an evidence base for the potential benefits and harms of *ante mortem* interventions
6. Except when consent may reasonably and lawfully be presumed, explicit consent for use of *ante mortem* interventions should routinely be obtained from the patient, parent, or their SDM. a. Even if specific consent for an *ante mortem* intervention is not required, it is best practice to provide some information about the intervention to the patient, their family or SDM as part of routine communication about the donation process
7. Information about the following aspects of *ante mortem* interventions is relevant for healthcare professionals and patients, their parents or other SDMs when making decisions about use of potential interventions: a. The potential to cause the patient or family discomfort or harm b. The invasiveness of the intervention, meaning the level of physical intrusion in the body c. Whether the intervention is commonly used in critically ill patients in the ICU d. The extent to which the intervention may alter the usual end-of-life care process for the patient e. The likely impact of the intervention on the probability of successful donation and transplantation of organs
8. The following information is important in guiding decision-making about use of ante mortem interventions in pcDCDD where these are lawful and clinically indicated: a. The known or estimated importance of achieving successful donation and transplantation to the child, parents, or SDM b. The importance of the child’s other known or estimated end-of-life goals that may be impacted by use of specific *ante mortem* interventions c. The importance of the patient’s family’s other goals regarding the end-of-life experience that may be impacted by use of specific *ante mortem* interventions

pcDCDD, Paediatric controlled donation after circulatory determination of death; SDM, Substitute decision maker.

**TABLE 8:** Consensus recommendations for end-of-life care in the context of pcDCDD.

1. Clinical management should be viewed as a continuum of care that includes family support throughout the donation process and appropriate comfort measures for patients who are potential pcDCDD donors
2. Supportive treatments should be maintained in patients potentially suitable for pcDCDD until donation can be discussed with the patient, parent, or SDM, unless medical suitability for pcDCDD is already excluded
3. The clinical approach to end-of-life care prior to and during WLSM should be consistent with that of routine end-of-life care in the absence of pcDCDD including provision of comfort care (e.g., sedatives and analgesia) a. The WLSM process that includes cessation of cardio-respiratory support and provision of comfort care (e.g., sedatives and analgesia) should be undertaken by the treating healthcare professionals and not organ donation staff overseeing or involved in the donation process b. Where required, analgesia and/or sedation should be provided for *ante mortem* interventions for donation as they would be for the same interventions if performed for therapeutic purposes
4. The clinical approach to end-of-life care prior to and during WLSM should optimise the likelihood of successful donation of organs for transplantation except when this may negatively impact the comfort of the patient. a. The location of WLSM should optimise quality of end-of-life care for the child and their family and/or SDM, as well as the success of organ recovery and utilisation of donated organs b. Where possible, the timing of WLSM should allow for optimal completion of the necessary donor assessment and preparation for the donation surgery c. Family members should always be offered the option of being present during WLSM and until death is determined
5. Prospective studies investigating the dying process following WLSM in paediatric populations (including neonates and infants) are needed

pcDCDD, Paediatric controlled donation after circulatory determination of death; WLSM, Withdrawal of life sustaining medical treatment; SDM, Substitute decision maker

The panel notably achieved consensus on the following definitions of a donor in the context of pcDCDD, while recognizing global variation in these definitions, particularly when adolescents are transferred to adult centres.

- Paediatric donor: any infant, child or adolescent aged ≥37 weeks CGA and <18 years.
- Neonate: refers to a child aged >37 weeks of corrected gestational age (CGA) and <28 days old.
- Infant: refers a child aged ≥28 days, <1 year old.
- Adolescent: refers to a child aged 13–17 years.

## Discussion

The first international meeting addressing paediatric deceased donation was organized by The Transplantation Society (TTS) in Geneva (Switzerland) in 2014, where discussions were held on the routine provision of opportunities for deceased donation by paediatric patients and a call was made for the development of evidence-based resources to inform best practices in deceased donation for neonates and children [20]. However, while pcDCDD has been addressed in some general DCDD guidelines [18, 21, 22] and in a small number of paediatric specific national guidelines [15, 16, 18], the present Project provided the first opportunity for experts from multiple countries and regions to develop consensus recommendations focused specifically on pcDCDD. Consensus was achieved on several key principles, reflecting a shared commitment to fundamental values that underpin end-of-life care for children and their families.

### Baseline requirements for pcDCDD programs

Successful implementation of pcDCDD programs requires multiple foundational elements. Key components include, but are not limited to, an enabling legal withdrawal of life sustaining measures (WLSM) framework, structured clinical education, accountable referral and coordination processes, and sustained governance and funding mechanisms.

It is critical to have a legal framework that allows for the WLSM, and the determination of death based on circulatory criteria in children (see Table 2). Without such legal clarity, no pcDCDD program or policy and procedure can safely develop or be sustained, as healthcare professionals require a legal mandate to perform pcDCDD. A legal framework is also essential for public trust in end-of-life care and donation activities.

A legal mandate is necessary for policy and procedure but is not sufficient for successful pcDCDD programs. While we do not have detailed international pediatric donor audit information to truly evaluate referral and consent performance, low rates in many countries with pcDCDD programs suggest that implementation of pDCDD remains a challenge (Figure 1). Development of programs requires ongoing education of healthcare professionals caring for paediatric patients (physicians, nursing, respiratory therapy, pharmacists, child life specialist, spiritual support, social workers and other members of the multidisciplinary team) to ensure that donation opportunities are offered to families in a timely and sensitive way. All healthcare professionals need regular reminders about the importance of identification and referral, especially because donation opportunities are infrequent and the potential benefits for donor families are impossible without referral [1, 12]. Ongoing communication between donor coordinators (DCs) or the staff of organ procurement organizations (OPO), intensive care teams and transplant programs should be established to ensure that referral criteria are consistent with surgical capacity and expertise. These logistic and implementation challenges require consistent funding and clear governance models.

### Avoiding missed opportunities for pcDCDD

The importance of designing clinical protocols to avoid missed opportunities for pcDCDD was evident in strong consensus favouring the establishment of clinical triggers for identification and referral of all potential pcDCDD donors (Table 3). Universal identification and referral of patients who are potential deceased organ donors is well documented as a key aspect of a successful system [23, 24]. This is particularly relevant in paediatrics, where potential donors are less frequent and therefore missed opportunities more critical [12, 18]. The precise clinical triggers for referral can vary slightly by jurisdiction but generally apply to any child for whom withdrawal of mechanical ventilatory or circulatory support is anticipated. All healthcare professionals who care for such children should be educated about the specifics of those triggers and empowered to refer when appropriate.

Surveys of intensive care adult and paediatric intensive care physicians suggest that they sometimes choose not to offer donation based on misconceptions around donor eligibility or assumptions regarding the family’s likelihood of consent/authorization [10, 25, 26]. Related surveys also showed that many healthcare professionals are not aware of legal requirements requiring referral, emphasizing the need for education around the implications of clinical triggers [10, 27]. Making referral a routine consideration for healthcare professionals during WLSM planning allows for normalization of donation as part of end-of-life care, which in turn ensures that no family who may have wished to pursue donation is denied that opportunity. Involvement of palliative care specialists can be beneficial when planning WLSM. Local protocols should be established regarding who contacts the family regarding donation and at what stage of the process that respects local resources and legal framework.

### Evaluation of medical suitability for pcDCDD

There is wide variability in the utilization of organs obtained via pcDCDD [5, 28]. This is in part due to substantive variation in the eligibility criteria and the diverse age range for paediatric donors, which in turn are likely influenced by local resources and capacity to transplant organs from paediatric donors with particular characteristics (see Box 1). Some programs transplant kidneys from pcDCDD donor infants as small as 1.9 kg [29] whereas others will only consider children over 5–10 kg [30]. While most common in the neonatal population, lower rates of pcDCDD organ utilisation have been reported in all age groups [13, 31]. This can lead to frustrating situations when families agree to donation, but perceived technical difficulties and surgical expertise regarding donor size or a lack of compatible recipients preclude donation. It is therefore important to have clear criteria that define medical suitability and priorities for pcDCDD that are implemented by local resources with available medical and surgical expertise. For example, capacity for donation of paediatric hearts following pcDCDD should be developed, due to the severity of unmet needs for heart transplantation in the paediatric population and the difficulties of size matching [32, 33]. Evaluations of donor potential and medical suitability in individual cases should strive to identify modifiable factors that may be limiting opportunities for pcDCDD. Medical suitability decisions about patients who may be considered “borderline” – for example, just above or below weight limit thresholds–are complex and will likely vary based on expertise of the medical and surgical transplant team [30].

Similarly, predictions around time to death are imprecise in patients undergoing planned WLSM [34], and very little data are available regarding such prognostication in paediatric patients [34–36]. Physician assessment or use of clinical tools based on patient factors are informative, but even in adults negative and positive predictive values remain around 80%–85% even with the best current models [34]. Thus it is often difficult to predict which children are likely to die following WLSM in a time frame that will enable successful recovery of organs for transplantation via the pcDCDD pathway [37]. The combined uncertainty around time to death and organ utilization can make transplanters and donation system administrators reluctant to invest the substantial resources required to pursue potential pcDCDD cases when the process might not end in successful transplantation. Despite fewer pcDCDD cases, this should not be a deterrent as similar concerns exist for DCDD in adults. Research is underway to improve prediction of death tools [38] in adults and eventually paediatric patients and international transplant outcome reporting should be encouraged to better define characteristics associated with favourable graft and patient outcomes from the pcDCDD pathway.

### The approach to communication about opportunities for pcDCDD

Several consensus recommendations focus on the adequate timing and approaches to informing patients, where appropriate and possible, their parents or other substitute decision-makers, about opportunities for pcDCDD (see Table 5). Organ donation is rare in children and many paediatric healthcare professionals lack confidence or expertise in donation. Organ donation discussions require trained donation personnel to assist with the donation process though the timely availability of that personnel may be limited due to resource constraints.

Evidence suggests that involving donation professionals increases consent/authorization rates for paediatric deceased donation [14, 26]. Potential conflict of interest about WLSM and donation issues has raised concerns that decisions may be influenced by donation professionals [39]. While there was strong consensus that decisions about donation should be separate from conversations and decisions about WLSM, countries may have different protocols for decision-making, diversity in laws governing donation, health system resources, and the responsibilities of donation personnel in these situations. Complete decoupling of end-of-life and donation decisions is not always possible. For example, families may mention the possibility of donation before final decisions about WLSM have been made. The recommendations are consistent with existing guidance that emphasises clear communication with families that focuses on the ongoing goals of treatment while discussing donation when it becomes a possibility or is raised by the family [17].

### Decisions about pcDCDD

How to incorporate a child’s previously expressed intentions or preferences in making formal decisions about donation opportunities has been a focus of considerable legal and ethical analysis [17, 40, 41]. The legal age of consent/authorization for medical treatment decision-making varies between jurisdictions and may differ from the age of consent/authorization for donation decisions. Some countries include the concept of “emancipated” or “mature” minors who may in specific circumstances be permitted to consent to medical procedures or to refuse life sustaining interventions if they are deemed to have the mental capacity and maturity to make such decisions [40–42]. When supporting decision-making about pcDCDD, healthcare professionals should be aware of relevant legal considerations in their jurisdiction, and ensure that a child’s legal rights are respected. This may entail respecting a child’s decision about WLSM and/or donation; more commonly it may require giving careful consideration to a child’s known or presumed values and preferences with regards to end-of-life care and donation.

There was consensus (see Table 6) that, when it is lawful to do so, a competent child’s expressed values and preferences should be respected where possible in decision-making about end-of-life care and donation. However, there was also consensus that a child’s parents or substitute decision maker (SDM) should make decisions about end-of-life care based on what they believe would be best for the child, all things considered. This raises the possibility of conflicts if a child’s known wishes are judged to be inconsistent with what lawful decision-makers believe would be best for the child. It is possible but unclear if panellists associated the latter recommendation with decision-making for children who had not expressed or were not competent to have made informed wishes regarding end-of-life care. Any potential contradiction and confusion is nevertheless resolved by the recommendation that a competent child’s known or presumed decision should be respected even if this conflicts with what SDMs believe is best for the child, provided such an approach is lawful.

Where the law does not mandate consideration of a child’s views, parents or SDM should nevertheless be encouraged to consider any known or estimated wishes regarding donation into account [17, 26, 43, 44]. The weight given to any expressed preference or the values of a minor child will depend on several factors, including the maturity of the child, the child’s intellectual capacity, and the clarity of the expressed intention [45]. Most children, even adolescents who may have legal capacity to consent to medical decisions, will likely never have expressed an intent regarding deceased organ donation, and estimation of their preferences may not always be possible especially in the case of younger children.

#### Ensuring quality of end-of-life care in the context of pcDCDD

A concern regarding pcDCDD has been that the logistics of the donation process can negatively impact end-of-life care [39, 46]. Changes to the location and process of WLSM can be required for pcDCDD to be feasible, or to reduce the risk of ischemic damage to organs intended for transplantation. At a minimum, the family will be separated from the patient immediately after the determination of death, so that the surgical recovery of organs can commence as swiftly as possible. Some commentators have questioned whether this inevitable separation makes pcDCDD ethically problematic, as the burden of changes to end-of-life care are perceived to fall on the patient and their family while the medical benefits accrue to transplant recipients [46]. However, this perspective may overlook the potential benefits that families and in some cases children may experience through the opportunity to pursue pcDCDD [47, 48]. While paediatric specific data are limited, the ability to perform an altruistic act at the time of devastating loss may be a valuable for many families, who may benefit psychologically, especially when donation is successful [49, 50].

Furthermore, changes to the location or even timing of WLSM are not inevitably problematic; even where changes necessitated by donation may be considered, a compromise on preferences that would otherwise be upheld, families (or patients, where relevant) may decide to proceed with pcDCDD because they believe the potential benefits will outweigh potential burdens. Ensuring that decision-makers are empowered to make informed decisions about end-of-life care and about donation is essential for ethical practice in pcDCDD, as indicated by recommendations in Tables 5, 6.

Underpinning many of the recommendations, and made explicit in those summarised in Table 8, is the principle that pcDCDD should be considered within the context of end-of-life care rather than in isolation. Consistent with a growing emphasis in the wider literature that donation should be a routine part of end-of-life care [26, 51], there was consensus that donation should be part of a continuum of end-of-life care, and that the clinical approach to end-of-life care when pcDCDD is planned should be aligned with routine care prior to and during WLSM. The recommendations encourage an integrative approach to pcDCDD; rather than assuming that tensions between donation and goals of care are inevitable, any disruptions to routine care that are necessary for donation and for which consent/authorization is provided should be minimised to the extent possible, and the comfort and wellbeing of the patient and family remain priorities.

### Use of ante-mortem interventions for pcDCDD

Similar to adult DCDD processes, some *ante mortem* interventions will be required for completion of any pcDCDD process. The broader consensus project defined such interventions as *“Any clinical procedure or test that is performed before death for the purpose of organ or tissue donation and transplantation, which would not occur in the absence of consideration of donation.”* [19] Blood draws and radiologic examinations are necessary to determine donor and organ eligibility and recipient compatibility, and the timing and location of WLSM will change to accommodate pcDCDD logistics [18]; heparin administration during WLSM, more invasive investigations may also be requested by transplant teams, such as biopsies or bronchoscopy. These interventions have often been implemented based on an inferences from physiologic principles, though few have been rigorously evaluated in high quality studies and even fewer in paediatric patients [39, 52, 53]. There was consensus regarding the need for research to provide evidence regarding the potential benefits and harms of *ante mortem* interventions in pcDCDD (see Table 7), and a recommendation that interventions should only be considered where there is a clear clinical justification for their potential to optimise donation and transplantation. Research will inform the broader questions of which investigations or interventions should be routinely recommended and help to support decision-making about their use.

Consensus was not sought on a defined set of ethically acceptable interventions for use in pcDCDD, nor did we seek to specify interventions for which consent may or may be presumed, as the risks and potential benefits of particular interventions may change over time and legal definitions of *ante mortem* interventions or consent requirements vary between jurisdictions [54]. Instead, consensus was pursued on the principles that should guide the approach to evaluation of and decision-making about use of *ante mortem* interventions for pcDCDD in any jurisdiction.

The importance of informed decision-making about use of *ante mortem* interventions in pcDCDD is evident in the recommendations outlined in Table 7. The limitations of the available evidence regarding interventions for pcDCDD were also recognized in the recommendation for research. While there was agreement that consent for interventions may sometimes be reasonably and lawfully presumed, it was recommended that even if specific consent is not required, best practice involves keeping families informed about all aspects of donation processes. This approach is also consistent with routine end-of-life care, where patients and/or their families are regularly updated on what is happening and why, even when explicit consent is not sought for interventions such as repeated blood tests or administration of medications. The recommendations are aligned with previous work that emphasises the importance of tailoring information to the preferences of parents or SDM, while respecting legal requirements [43, 55, 56].

Several considerations were identified as relevant for informed decision-making about use of *ante mortem* interventions in pcDCDD (see Table 7). Information about the child’s known or estimated preferences and goals with regards to end-of-life care and donation was considered important in guiding decision-making where possible, consistent with the aforementioned recommendations regarding end-of-life care and donation decision-making in the context of pcDCDD (see Table 6). The possibility of achieving a child’s known or estimated donation goals or those of their family is evidently a key potential benefit of using *ante mortem* interventions that may balance concerns about the potential burdens of some interventions [43].

While the recommendations in this paper are largely consistent with those reported in the context of the adult cDCDD pathway [57], some ethical considerations may be more complex in the paediatric setting. When compared to adults, children’s goals or preferences with regards to donation or end-of-life care, let alone *ante mortem* interventions, are far less likely to be known or able to be inferred depending on the age of the child. Reliance on a ‘best interests’ approach to decision-making rather than making decisions based on the potential donor’s known or inferred preferences is not unique to cDCDD in the paediatric setting, however, parental beliefs, familial interests, and decisions and interest of the child can create challenges and complicate decisions. For example, some authors have cautioned against the possibility that grieving parents may consent to *ante mortem* interventions that are unduly burdensome for the child in order to improve the possibility of donation [58]. Conversely, some parents might be unwilling to agree to interventions of negligible burden or risk, or pcDCDD more generally, if they believe the impact of the donation process may be burdensome [43]. Clear guidance for parents and SDM is required as they remain responsible and must agree to decisions regarding end-of-life care and organ donation.

### Limitations

While robust, the methodology of this Consensus Project has several limitations, as outlined elsewhere [19]. Although the consensus achieved by this diverse panel of experts suggests a reassuring level of agreement at the principled level internationally, a degree of bias is inevitable. Paediatric deceased donation, and pcDCDD in particular, is a relatively small field of activity with limited available evidence to support best practices or to inform the estimation of values and preferences of various stakeholders. Consequently, some of the recommendations outlined herein may be difficult to translate into policy and practice in some contexts or may lack the specificity to provide substantive guidance where some changes are needed. Resource limitation and cultural considerations can clearly impact individual jurisdictions for pcDCDD and must be considered.

## Conclusion

Although there is limited quality evidence available the recommendations of this Consensus Project provide a foundation for pcDCDD that can be adapted to local, legal, and regulatory systems to improve global access to pcDCDD. Legal, financial, cultural, religious and a multitude of other factors will influence the capacity of a healthcare system to create and maintain a robust donation program and these recommendations cannot address the nuances of those various realities. In one country, a lack of cultural acceptance of WLSM may be the largest barrier to implementation of pcDCDD, whereas the principal barrier in another country may be the lack of sufficient surgical expertise to utilise the organs from small pcDCDD donors.

pcDCDD is a rare but impactful event that provides life-saving transplants for other children and adults [2]. The recommendations produced by this Consensus Project provide valuable guidance for healthcare professionals and policymakers seeking to establish or improve pcDCDD programs. Many of the recommendations may also have relevance to paediatric DNDD, which remains an important donation pathway. However, more knowledge is needed to support ongoing efforts at the international and local levels to increase and improve outcomes of pcDCDD. The global paediatric donation community must continue to invest in and communicate rigorous and multidisciplinary evaluations of pcDCDD programs to improve knowledge of medical suitability criteria, effective strategies to support decision-making, outcomes of ante mortem intervention use, and several other factors that influence experiences and outcomes of pcDCDD. This research and evaluation is necessary to ensure that all families can be offered the opportunity and ensure that healthcare teams incorporate organ donation as an integral part of end-of-life care.

Box 1 **Factors influencing opportunities for pcDCDD.**

1. Clinical characteristics of the potential paediatric donor that may influence the duration of time between WLSM and circulatory arrest, and hence functional warm ischaemic time (FWIT), include: a. Presence and severity of a devastating brain injury, including depth of coma and number of absent brainstem reflexes.
  b. Airway patency (e.g., airway pathology, sleep apnoea, obesity).
  c. Dependence on mechanical ventilation and oxygen requirements prior to WLSM.
  d. Dependence on vasoactive and/or mechanical circulatory supports.
2. Several local healthcare system factors and choices made by patients, their parents or other SDM regarding end-of-life care may influence the likely duration of acceptable FWIT and cold ischaemic times in the setting of cDCDD. These include:
  a. End-of-life care practices, including the mode of WLSM (e.g., extubation), the approach to provision of comfort care with intravenous sedatives or analgesic agents (e.g., use of deep sedation versus the minimal amount for symptom relief).
  b. Location of WLSM and consequent time required to move the deceased person to the operating room.
  c. Availability and use of various *ante mortem* interventions.
  d. Availability and use of machine perfusion technologies, including *ex situ* organ perfusion and *in situ* normothermic regional perfusion (NRP).
  e. Availability of surgical expertise and operating theatre access.
3. Additional factors that may influence decisions to attempt pcDCDD in specific cases include:
  a. Characteristics of the potential donor that may influence the utilisation or likely outcomes of transplantation if an organ(s) is recovered, e.g., very young age, small size, or non-standard risk for infectious disease transmission.
  b. Relevant transplant unit acceptance practices (e.g., ability to transplant extended criteria donated organs into suitable candidates, noting practices will vary for different organs).
  c. Travel distances and transport options for recovery teams, organ donation staff, and recovered organs.

pcDCDD Paediatric controlled donation after circulatory determination of death.

WLSM Withdrawal of life sustaining medical treatment.

SDM Substitute decision maker.

FWIT Functional warm ischemic time.

## Data Availability

The raw data supporting the conclusions of this article will be made available by the authors, without undue reservation.
